# First-Principles Calculations and PMUT Applications of Piezoelectric Thin-Film Materials

**DOI:** 10.3390/mi17030377

**Published:** 2026-03-20

**Authors:** Chengwei Che, Shanqing Yi, Caishuo Zhang, Xinyi Zheng, Xingli He, Dacheng Xu

**Affiliations:** School of Electronic and Information Engineering, Soochow University, Suzhou 215006, China

**Keywords:** PMUTs, first-principles calculations, analytical acoustic-field model, large-array topologies, PZT, ScAlN

## Abstract

High-performance piezoelectric micromachined ultrasonic transducers (PMUTs) are crucial for portable medical imaging and sensing. The efficiency of advanced PMUTs relies on high-quality piezoelectric thin films and optimized device designs. However, variability in common piezoelectric thin films like Sc_x_Al_1−x_N (ScAlN) and PbZr_1−x_Ti_x_O_3_ (PZT) often leads to inaccurate material parameters—especially those derived from thick ceramics. To enhance simulation accuracy in standard designs affected by these inconsistencies, this work introduces an optimization framework combining first-principles calculations with multiphysics simulations. First, the intrinsic properties of PZT and ScAlN are analyzed through atomistic calculations, confirming that PZT, with its higher electromechanical coupling coefficient, is better suited for actuation. The parameters obtained from these calculations calibrate the finite-element model, addressing issues of missing or inaccurate data in commercial software libraries. Next, an efficient analytical acoustic-field model is developed. Compared to full-wave simulations in COMSOL, this model significantly reduces computational cost while maintaining accuracy, allowing for quicker scanning and optimization of large-array topologies. Additionally, results demonstrate that each individual hexagonal PMUT element outperforms a comparable circular element, achieving a peak SPL of 90.4 dB at 4.9 MHz versus 89.7 dB at 2.8 MHz. This higher acoustic output and operating frequency enable improved spatial resolution and sensitivity. This modeling approach, based on intrinsic material properties, provides a solid theoretical foundation for designing high-precision, low-power ultrasonic devices.

## 1. Introduction

With the rapid development of Micro-Electro-Mechanical Systems (MEMS) technology, piezoelectric thin films are increasingly used in a wide range of applications, from RF filters and energy harvesters to high-precision sensors and actuators [1]. Building on this foundation, their use in medical ultrasound has become especially transformative. Ultrasound is now a crucial tool for clinical diagnosis and continuous monitoring because it is non-ionizing, offers strong real-time capabilities, is relatively affordable, and is suitable for point-of-care settings. It continues to improve in areas like hemodynamic assessment, including vascular wall motion tracking and pulse wave velocity (PWV) [2,3,4]. As systems move toward portable and wearable designs, MEMS-based miniaturized ultrasonic transducers support device miniaturization and low-power operation. Notably, piezoelectric micromachined ultrasonic transducers (PMUTs) have gained significant attention due to their compatibility with array integration, low drive voltages, and potential for system-level integration [5]. However, designing high-performance PMUT arrays presents a multiscale, multiphysics challenge: intrinsic material tensors influence electromechanical coupling; membrane structural modes affect displacement, bandwidth, and ringing; and the array’s acoustic field—including the main lobe, sidelobes, and grating lobes—directly impacts the separability of wall echoes and the accuracy of time-delay estimation, which in turn influences the measurement accuracy of PWV and related parameters [3,4,5,6].

Existing PMUT optimization efforts mainly target geometric design and process enhancements—such as piezoelectric layer stacking, membrane structures, and electrode configurations—to improve sensitivity or effective bandwidth [5,6,7]. For circular-membrane PMUTs, residual stress and boundary conditions can greatly affect membrane deflection, resonance, and pulse–echo performance. Additionally, adding slots or other “stress-relief/damping-enhancement” features to the membrane or silicon layer can lower the Q factor and reduce sensitivity to residual stress, thereby increasing ringing and effective bandwidth [8,9]. However, most device models still depend on macroscopic or empirical material parameters, neglecting how microstructural and compositional variations in thin films can change their intrinsic tensors—leading to uncertainties in modeling accuracy [5,6,7,8].

The performance of PMUTs heavily relies on the properties of piezoelectric thin-film materials, making research into these characteristics essential for developing high-performance PMUTs. Studies have examined the piezoelectricity of PZT systems and phase-transition mechanisms from first principles, providing the theoretical foundation for intrinsic material tensors [10,11,12]. Daoust et al. used density-functional perturbation theory (DFPT) to calculate the composition-dependent piezoelectric, elastic, and dielectric properties of Sc- and other-doped AlN alloys, supplying traceable material inputs for thin-film piezoelectric devices [13]. However, these studies mainly focus on the material level; comparatively fewer works incorporate intrinsic tensors into device-level multiphysics modeling of PMUTs and extend the framework to array acoustic fields and topology optimization.

The fabrication and system validation of row–column-addressed PZT-based PMUT arrays have been documented, demonstrating the engineering feasibility of array implementation [14,15], where bulky PZT (PZT-5A or PZT-5H) and sol–gel-processed PZT were used in parallel. Jensen’s spatial impulse response theory and the Field II framework provide efficient semi-analytical predictions of array acoustic fields, while Hlawitschka and McGough introduced fast beam prediction methods for regular arrays to reduce the computational load for large apertures [16,17,18,19]. However, the former is not ideal for rapid, large-scale scans across array topology and pitch, whereas the latter generally lacks a consistent unit-source model coupled with intrinsic material parameters. Therefore, a unified modeling chain that covers materials, devices, and arrays remains essential.

Motivated by these considerations, this work presents an integrated modeling and optimization approach that links materials, devices, and arrays. First, DFT (density-functional theory) and DFPT are used to compute the intrinsic piezoelectric, elastic, and dielectric tensors of PZT and Sc_0.25_Al_0.75_N as material inputs [10,11,12,13]. Second, a semi-analytical array acoustic-field model based on the Rayleigh integral and the spatial impulse response concept is developed to efficiently evaluate array directivity and grating-lobe patterns [16,17,18,19]. Finally, the results are validated through COMSOL Multiphysics (version 6.2) simulations, and the influence of microscopic material-parameter variations on macroscopic acoustic performance and array design trade-offs is examined. This work offers reusable theoretical and computational tools for fast, application-specific design of high-performance PMUT arrays. As this study is entirely computational, the proposed parameters and design insights provide a foundation that can be further validated and calibrated against experimental data in future work.

## 2. First-Principles Calculations of Piezoelectric Materials

To obtain accurate material parameters needed for finite-element simulations of PMUTs, this study uses DFT-based first-principles calculations to systematically analyze the crystal structures and multiphysics coupling properties of tetragonal-phase PZT and wurtzite ScAlN. All calculations are performed using the VASP (Vienna Ab initio Simulation Package) code [20]. The critical piezoelectric stress constant *e*_33_ is defined by the following formula:

(1) $$e_{33}=e_{33}^{\text{clamped-ion}}+\frac{4eZ^{*}}{\sqrt{3}{a_{0}}^{2}}\frac{du}{dc}.$$

Here, $e_{33}^{clmaped\text{-}ion}$ is the “clamped-ion” contribution, Z* is the axial component of the dynamical Born effective charge tensor, *a*_0_ is the equilibrium lattice parameter, and *du/dc* is the sensitivity of the internal parameter to strain along the crystallographic axis.

### 2.1. Computational Methods and Parameter Settings

The calculations used the Perdew–Burke–Ernzerhof (PBE) functional within the generalized gradient approximation (GGA) [21] to describe exchange-correlation effects, and the interaction between ionic cores and valence electrons was handled with the projector augmented-wave (PAW) method [22,23]. To balance accuracy and computational efficiency, a uniform plane-wave cutoff energy of 600 eV was applied. In the self-consistent field (SCF) cycle, the electronic energy convergence criterion was set to 1 × 10^−6^ eV; during structural optimization, the force convergence threshold for ionic relaxation was set to 0.02 eV/Å.

For the Pb(Zr,Ti)O_3_ (PZT) system, the Pb_d pseudopotential, which treats the 5d electrons as valence electrons, was used to accurately describe the hybridization between the Pb 5d states and O 2p states. For Zr and Ti, pseudopotentials that include the semicore electrons, specifically Zr_sv (4s/4p) and Ti_sv (3p), were employed. These semicore states play a significant role in orbital hybridization and the polarization response between the transition metals and oxygen; including them as valence electrons improves the accuracy of the calculated elastic constants and piezoelectric coefficients. For the ScAlN system, the standard Al pseudopotential (considering 3s^2^3p^1^) and the N pseudopotential (2s^2^2p^3^) were used. Sc was modeled with the Sc_sv pseudopotential, which includes the 3p semicore states, to better capture the covalent character and polarization behavior of the Sc–N bonds. All pseudopotentials were obtained from the PAW_PBE potential set recommended within VASP.

Because of the wurtzite structure of ScAlN (space group P6_3_mc), which has well-defined hexagonal symmetry, symmetry constraints were applied during the calculations (ISYM = 2) to speed up computation. Post-processing of the self-consistent field results confirmed that the stress-strain relationships conform to the constraints of the hexagonal crystal system. For PZT, considering the potential for local distortions like oxygen octahedral tilting and cation disorder near the morphotropic phase boundary, the actual ground-state symmetry may differ from the ideal tetragonal structure. Therefore, symmetry constraints were disabled (ISYM = 0) during both structural relaxation and elastic constant calculations, allowing atomic positions and the cell shape to fully relax. This method was used to account for possible subtle structural distortions impacting the elastic properties. Although the relaxed unit cell remained nearly tetragonal (a ≈ b ≠ c), the atomic positions were no longer strictly constrained by symmetry.

**Figure 1:**
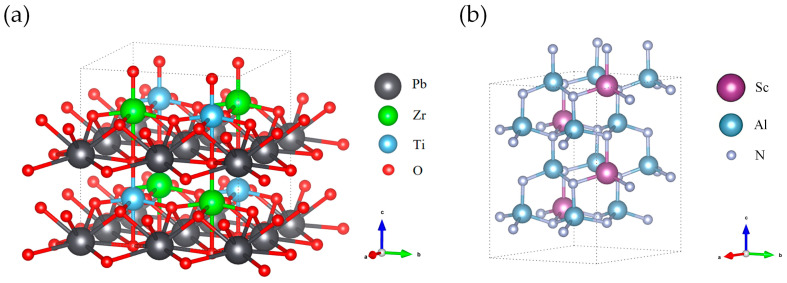
Schematic diagram of the unit cell structure of tetragonal PZT (**a**) and wurtzite ScAlN (**b**) for DFPT calculations.

After obtaining the stable ground-state structures, DFPT [24] was used to calculate the elastic stiffness tensor (*C_ij_*), piezoelectric stress tensor (*e_ij_*), and dielectric permittivity tensor (*ε_ij_*). By evaluating the second derivatives of the total energy with respect to external perturbations (e.g., lattice strain or an applied electric field), DFPT enables the accurate determination of the full tensor properties, including internal-strain (internal-displacement) contributions. The unit cell structures of PZT and ScAlN used for calculations are shown in Figure 1.

### 2.2. Tetragonal-Phase PZT Model and Properties

PZT exhibits optimal piezoelectric performance near the morphotropic phase boundary (MPB). To simulate the structural characteristics in the vicinity of the MPB, this study constructed a tetragonal Pb(Zr_0.5_Ti_0.5_)O_3_ supercell with a Zr/Ti ratio of 50/50. The model was built by expanding a 2 × 2 × 2 PbTiO_3_ unit cell and contains 40 atoms (8 Pb, 4 Zr, 4 Ti, and 24 O). To obtain the thermodynamically most stable configuration, Zr and Ti atoms were arranged in an alternating pattern. Brillouin-zone integrations were performed using a 3 × 3 × 3 *Γ*-centered *k*-point mesh.

After structural relaxation, the lattice constants were *a* = *b* = 8.089 Å and *c* = 8.246 Å, indicating a notable tetragonal distortion. The elastic stiffness matrix, piezoelectric stress matrix, and relative dielectric permittivity matrix calculated by DFPT are listed in Table 1, Table 2 and Table 3, respectively. The results show that PZT has a relatively low elastic modulus along the polarization direction (*C*_33_ = 95.23 GPa) and a high piezoelectric coefficient (*e*_33_ = 4.31 C/m^2^), which is advantageous for achieving highly sensitive acoustic-energy conversion.

The PZT supercell model with a Zr/Ti ratio of 50/50 created in this study aims to represent the composition near the morphotropic phase boundary, an area known for its excellent piezoelectric properties. The alternating pattern of Zr and Ti atoms used in the model shows an idealized ordered structure, chosen to achieve a thermodynamically stable setup suitable for subsequent DFPT calculations. However, in real polycrystalline PZT thin films, Zr and Ti are randomly distributed and may display local chemical inhomogeneity. As a result, the intrinsic tensors (such as elastic, piezoelectric, and dielectric constants) calculated from this ordered structure reflect an “averaged” response or the response of a specific configuration, which might not fully capture local lattice distortions and polarization fluctuations caused by cation disorder. Still, this approach effectively isolates the intrinsic “clamped-ion” and “internal-strain” contributions, excluding extrinsic factors like domain wall motion, and thus provides a straightforward, atomistic reference point for comparison with macroscopic ceramic properties. When using these parameters in device simulations, especially in comparison with “libraries” based on polycrystalline ceramics, it is important to recognize that the calculated values represent the intrinsic response of a single grain, while the “library” parameters naturally include the complex effects of polycrystalline averaging, defects, residual stress, and processing conditions.

### 2.3. Model and Properties of Sc-Doped AlN (Sc_x_Al_1−x_N)

Additionally, to evaluate the performance of high-stiffness piezoelectric materials, a 2 × 2 × 2 supercell (32 atoms) was constructed for the wurtzite Sc_x_Al_1−x_N alloy with 25% Sc. Accurate alloying was achieved by substituting Al with Sc at specific lattice sites and selecting the lowest-energy atomic configuration. Due to differences in lattice dimensions, a denser k-point mesh of 5 × 5 × 3 was used. The cell shape, volume, and atomic positions were fully relaxed using ISIF = 3, resulting in optimized lattice parameters of a = b = 6.4850 Å and c = 10.1419 Å.

The calculations (which were presented in Table 4, Table 5 and Table 6) show that Sc_0.25_Al_0.75_N exhibits a high-stiffness characteristic, with *C*_33_ reaching 171.7 GPa, significantly exceeding that of tetragonal-phase PZT. When comparing the main properties of the two materials along the c-axis, Sc_0.25_Al_0.75_N has a lower piezoelectric coefficient than PZT; however, its higher acoustic velocity and potential for a higher mechanical quality factor make it better suited for high-frequency applications. Conversely, PZT—with its superior *e*_33_ and *ε*_33_—offers clear advantages for low-frequency, high-sensitivity sensing and actuation.

### 2.4. Validation of DFT Results

To verify the accuracy of the first-principles calculations (DFT-PBE) used in this work and to compare the performance of the emerging piezoelectric material Sc_0.25_Al_0.75_N with established materials, we conducted a detailed comparison of our calculated key parameters—elastic constant *C*_33_, piezoelectric stress coefficient *e*_33_, and relative dielectric permittivity *ε*_33_—against literature data for both Sc_0.25_Al_0.75_N and the conventional ferroelectric Pb(Zr_0.5_Ti_0.5_)O_3_.

As shown in Table 7, the computational results for ScAlN in this study align well with the literature. The calculated *C*_33_ for Sc_0.25_Al_0.75_N is approximately 170 GPa, indicating the “elastic softening” effect caused by Sc doping—a reduction of about 50% compared to approximately 345 GPa for single-crystalline AlN. Additionally, the calculated *e*_33_ (1.97 C/m^2^) also falls within a reasonable range, confirming the significant increase in piezoelectric response with 25% Sc doping. For PZT (50/50), the relatively lower dielectric constant and piezoelectric coefficient (compared to ceramic materials) verify that our model accurately captures the clamped-ion (ionic) contributions of the tetragonal lattice, excluding extrinsic domain-wall effects. In particular, the calculated *C*_33_ effectively reflects the softer mechanical framework typical of PZT, demonstrating the universality of the computational model across different crystal structures (wurtzite vs. perovskite).

## 3. PMUT Design and Modeling

**Figure 2:**
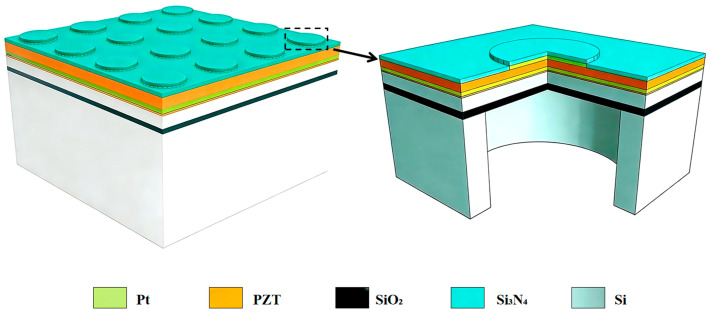
Schematic of the 4 × 4 array PMUT structure; the right panel shows a zoomed-in image of a single PMUT unit.

The calculated material parameters were used for PMUT simulations. Generally, the basic structure of a PMUT from top to bottom includes a top electrode, a piezoelectric layer, a bottom electrode, a structural (elastic) layer, and a substrate. To examine the performance of an arrayed PMUT, this work adopts a multilayer stack arranged from top to bottom as follows: silicon nitride, a platinum (Pt) top electrode layer, a PZT/ScAlN piezoelectric layer, a platinum (Pt) bottom electrode layer, a piezoelectric seed layer, single-crystal silicon (Si), and an insulating silicon dioxide (SiO_2_) layer. Three-dimensional schematics of the 4 × 4 array and a single unit cell of this multilayer structure are shown in Figure 2, with layer thicknesses from top to bottom of 0.2 μm, 0.2 μm, 0.5 μm, 0.2 μm, 0.03 μm, 2.2 μm, and 1.09 μm.

In most MEMS devices, a thin film composed of several layers can be approximated as a radially averaged circular plate. Under the small-deflection assumption, the mechanical equilibrium equation of a circular tensioned plate subjected to a uniform pressure *P* is given in [29] as follows:

(2) $$\nabla^{4}w-N^{\prime }\nabla^{2}w=P$$

where ∇^4^*w* describes the rate of curvature change of the membrane deflection. When multiplied by the membrane’s bending stiffness *D_m_*, it represents the internal resistance stress related to bending deformation. During deformation, the in-plane tension produces a transverse component, and the restoring force is proportional to ∇^2^*w*; that is, the combined bending restoring force and tension-induced restoring force balance the external pressure. The membrane tension per unit length *N*′ is derived from the product of tension per unit area *T* and the total thickness, reflecting the tensile stress in the membrane when it is pre-stretched (tensioned). The membrane tension *N*′, the areal tension *T*, and the bending stiffness *D_m_* are described by the following formulas [29,30]:

(3) $$N^{\prime }=T\cdot \sum_{i=1}^{N}h_{i},$$

(4) $$T=\frac{\sum_{i=1}^{N}T_{i}h_{i}}{\sum_{i=1}^{N}h_{i}},$$

(5) $$D_{m}=\frac{1}{3}\sum_{n=1}^{N}\frac{Y_{n}}{1-{\nu_{n}}^{2}}({\left(z_{n}-z\right)}^{3}-{\left(z_{n-1}-z\right)}^{3}),$$

where *h_i_* is the thickness of each layer, *Y_n_* is the Young’s modulus of each layer, *z* denotes the position of the neutral plane, and *z_n_* and *z_n_*_−1_ are the coordinates of the top and bottom surfaces of the n-th layer, respectively. For a circular plate subjected to a uniform load, by combining Equations (2)–(5) with the boundary conditions, the deflection profile solution and the first-order resonant frequency of a radially circular plate with radius *R* can be expressed as follows (clamped circular-plate eigen-solution/Bessel mode) [29]:

(6) $$w(r)=A(J_{0}(\lambda_{1}\frac{r}{a})-\frac{J_{0}(\lambda_{1})}{I_{0}(\lambda_{1})}I_{0}(\lambda_{1}\frac{r}{a}))e^{j\omega t},$$

(7) $$\omega ={\left(\frac{\lambda }{R}\right)}^{2}\sqrt{\frac{D_{m}}{\sum_{i=0}^{N}\rho_{i}h_{i}}},$$

where *A* is the amplitude coefficient; *J*_0_ and *I*_0_ are the 0th-order Bessel function and the 0th-order modified Bessel function, respectively; and *λ*_1_ is the eigenvalue of the first-order resonant mode. In acoustics, the acoustic-field output of a PMUT unit cell or array is often estimated using radiation-integral-based methods or an equivalent point-source superposition approach [31]. The operating PMUT can first be approximated as a resonant circular disk with a backing plate: the maximum displacement amplitude occurs at the disk center and decreases with radius to zero (due to the clamped boundary at the disk edge). Accordingly, the velocity distribution over the top surface of a circular PMUT device can be obtained as follows [29]:

(8) $$u(r)=\frac{\partial w(r)}{\partial t}=A\omega (J_{0}(k_{1}r)-\frac{J_{0}(k_{1}a)}{I_{0}(k_{1}a)}I_{0}(k_{1}r))e^{j\omega t}.$$

Accordingly, the acoustic pressure generated by the PMUT at an arbitrary point in the half-space in front of the device (in cylindrical coordinates, with radial distance *r* from the disk center and an angular deviation *θ* from the central axis) can be expressed as [31]:

(9) $$P=\iint j\frac{k_{2}\rho_{0}c_{o}}{2\pi h}A\omega (J_{0}(k_{1}r)-\frac{J_{0}(k_{1}a)}{I_{0}(k_{1}a)}I_{0}(k_{1}r))e^{j(\omega t-k_{2}h)}ds,$$

where *k*_2_ = *ω/c* is the wavenumber, *ρ*_0_ is the density of the acoustic medium, *c*_0_ is the sound speed in the medium, and *h* is the distance between a point source and the field point at which the sound pressure is evaluated. In the sound-pressure distribution, the source directivity is generally defined as the normalized angular response under the far-field approximation [31]:

(10) $$D(\theta )=\frac{P_{\theta }}{P_{\theta =0}},$$

where the angle *θ* is defined as the angle relative to the central axis when the observation distance is much larger than the source dimensions. Here, it is also required that the distance between the field point and the source be much larger than the source radius (far-field condition). Evidently, for both a single element and an array, the region where the acoustic field is most concentrated is typically located near the central axis. Therefore, when designing the acoustic output of a PMUT, primary focus is placed on the on-axis performance of the PMUT array. If only a disk-shaped PMUT element is considered, the sound pressure generated on its central axis at a distance *h* can be written as follows (axisymmetric integral) [31]:

(11) $$P=\int j\frac{k_{2}\rho_{0}c_{o}}{2\pi h}A\omega (J_{0}(k_{1}r)-\frac{J_{0}(k_{1}a)}{I_{0}(k_{1}a)}I_{0}(k_{1}r))e^{j(\omega t-k_{2}h)}2\pi rdr.$$

## 4. Simulation and Validation

### 4.1. Performance Comparison of Different Piezoelectric Materials

Based on the first-principles calculations above, this study initially compares the transmitting sensitivity of circular PMUTs with identical characteristic dimensions but different piezoelectric materials. Meanwhile, the Rayleigh distance is defined as *R*_0_ = *A*/*λ*, where *A* is the element area and *λ* is the wavelength, and when *s* >> *R*_0_, it can be considered as the far field. The element radius is 50 μm, and the acoustic medium is water with a density and sound velocity of 998 kg/m^3^ and 1481 m/s, respectively. The excitation voltage is 1 V, and the boundary of the water domain is set as a spherical radiation boundary condition to simulate the attenuation law in an infinite water domain. We systematically evaluate the frequency response of the sound pressure level (SPL,re 20 μPa) at an on-axis distance of 1 m, along with the on-axis sound-pressure attenuation, for arrays made of four piezoelectric materials: AlN, PZT (tetragonal Pb(Zr_0.5_Ti_0.5_)O_3_), PZT-5H, and Sc_0.25_Al_0.75_N.

**Figure 3:**
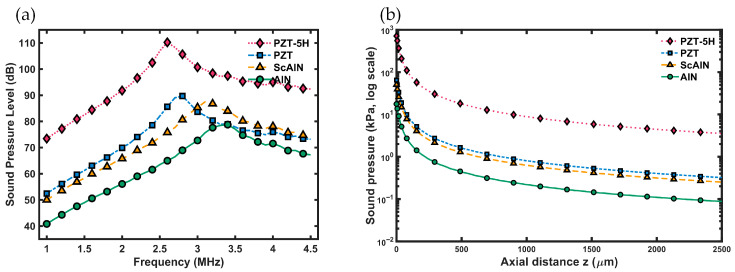
(**a**) On-axis SPL at 1 m for PMUTs made with different piezoelectric materials. (**b**) Log-scale plot of the on-axis sound-pressure output for PMUTs with various piezoelectric materials.

As shown in Figure 3a, all four material-based arrays display typical resonant-response behavior; however, their peak frequencies and maximum SPLs vary significantly. The PMUT based on PZT-5H provides the most remarkable acoustic output, reaching a peak SPL of 110.2 dB at 2.6 MHz—about 22.8% higher than tetragonal-PZT (89.7 dB at 2.8 MHz). The AlN array peaks at 78.8 dB at 3.4 MHz, with a relatively flat response curve. The Sc_0.25_Al_0.75_N array exhibits acoustic characteristics between those of AlN and PZT, with a peak of 87.5 dB at 3.1 MHz. At the highest frequency of 3.4 MHz, the corresponding minimum wavelength is 435.6 μm, and the Rayleigh distance is roughly 18 μm. Since *R*_0_ is much less than 1 m, all sound pressure values measured at 1 m are in the far-field region, complying with the spherical radiation wave attenuation law. The performance advantage of PZT-5H mainly stems from its higher piezoelectric constants and electromechanical coupling coefficient, which produce larger mechanical displacement and acoustic radiation pressure under the same electric-field excitation.

As shown in Figure 3b, the PMUT based on PZT-5H in COMSOL demonstrates the highest sound-pressure transmission sensitivity. However, the material parameters in the current COMSOL database mainly come from bulk piezoelectric ceramics and do not consider the crystal orientation and stress state typical of PZT thin film deposition by PVD processes. Using the library parameters directly can therefore cause significant differences between simulated results and the actual performance of thin-film devices. As a result, this work uses the intrinsic parameters obtained from DFT calculations as input. This method not only avoids uncertainties related to empirical parameters but also offers a more accurate physical basis for designing high-frequency PMUTs.

It is important to recognize that these DFT-derived parameters reflect the intrinsic response of a single crystal or an idealized ordered structure. While they provide a more physically consistent starting point than bulk ceramic libraries, they do not automatically include extrinsic effects common in thin films, such as residual stress, grain boundaries, or substrate clamping. Therefore, when applying these parameters in device simulation, consider them as a high-quality baseline that can be further refined with experimental data for a specific thin-film process.

To evaluate how such deviations might affect device performance, we performed a basic sensitivity analysis by changing the most critical parameter—the piezoelectric stress constant *e*_33_—by ±10% and ±20%. The resulting changes in the on-axis sound pressure level (SPL at 1 m) are summarized in Table 8. As shown, a 20% decrease in *e*_33_ causes about a 3.3 dB drop in SPL (the deviation of absolute SPL is less than 4%), indicating that the acoustic output is very sensitive to the film’s actual piezoelectric response. Conversely, a 20% increase results in a +2.3 dB rise. The sensitivity slightly exceeds the linear scaling prediction, reflecting the coupled electromechanical interactions modeled by the full multiphysics approach. This analysis stresses the importance of using a reliable intrinsic baseline (provided by DFT), while recognizing that final device performance also depends on the specific microstructure and stress conditions of the thin film. Therefore, the DFT parameters provide a solid physical foundation that can be further refined with experimental data from a specific deposition process.

### 4.2. Comparison Between Theory and Simulation

**Figure 4:**
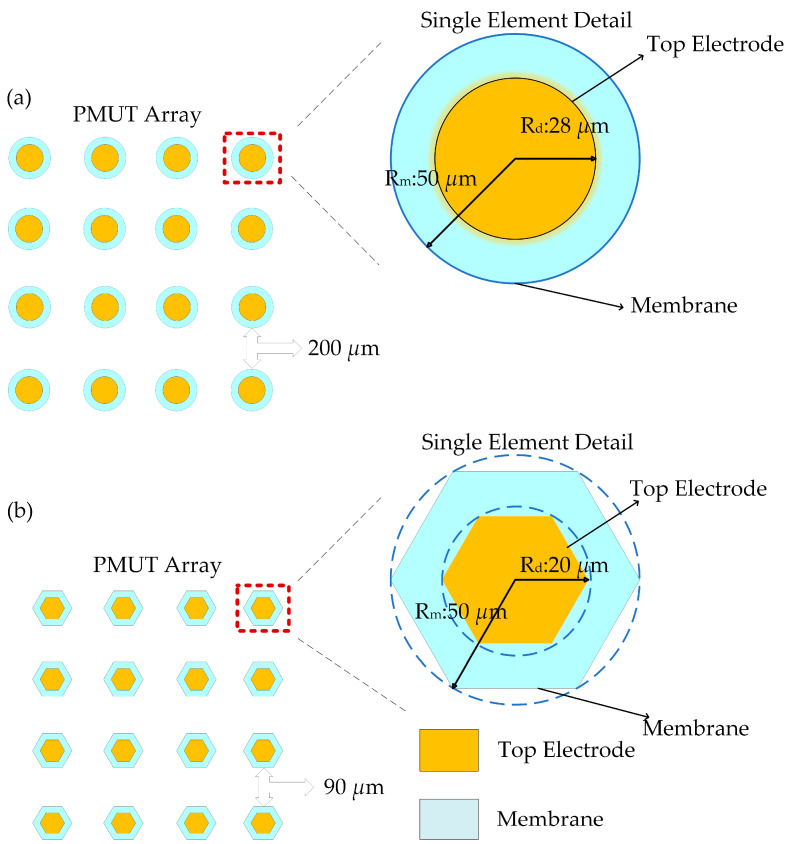
PMUT square array configurations, with each element featuring either a (**a**) circular or (**b**) hexagonal piezoelectric composite partly covered by the top electrode; the array elements are arranged at equal distances.

For simulation validation, an isolated element is used as the reference. As shown in Figure 4, arrays with circular and hexagonal units, featuring 2 × 2, 3 × 3, and 4 × 4 square configurations, are constructed sequentially. A systematic comparative analysis is then performed under the synchronous excitation of all elements in the array. Simulations are carried out using COMSOL Multiphysics with the “Acoustic–Piezoelectric Interaction, Frequency Domain” multiphysics interface within the Acoustics Module to develop a 3D finite-element model. By applying refined controls and coupling constraints across Solid Mechanics, Electrostatics, and Pressure Acoustics, the electromechanical–acoustic coupling effects are comprehensively captured. The acoustic domain (water) is modeled as a hemisphere with a radius of 2.5 mm. The acoustic domain is meshed with free tetrahedral elements, with the maximum element size set to *λ*/6, ensuring at least six elements per wavelength to accurately resolve wave propagation. The solid domain (PMUT multilayer membrane structure) is meshed using a swept mesh. A spherical-wave radiation boundary condition is applied to the outer surface of the hemisphere, assuming that the outgoing wave is spherical and perpendicular to the boundary, effectively absorbing outward-propagating waves to mimic an unbounded acoustic space. For time discretization, the transient solver uses the generalized-α method with a fixed CFL number of 0.05 to limit the discretization error from the time step.

**Figure 5:**
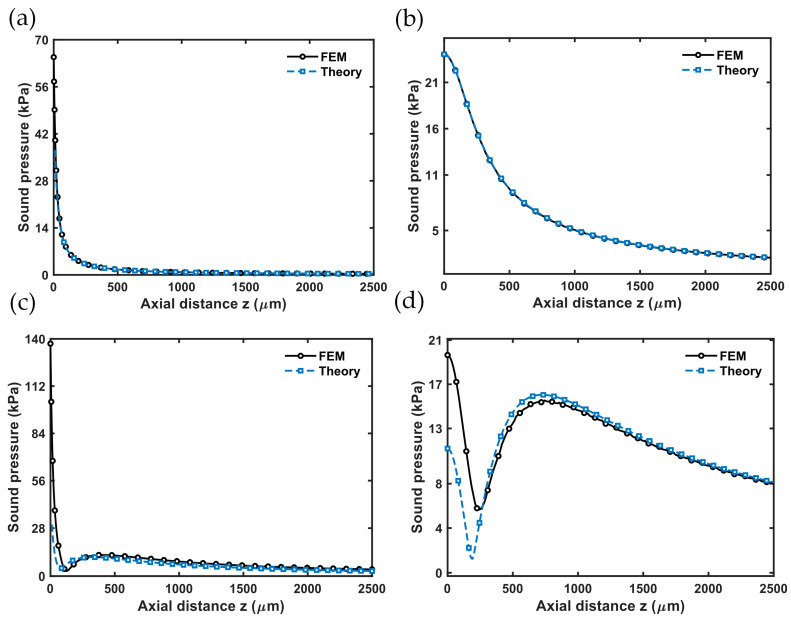
Variation of sound pressure along the acoustic axis for (**a**) 1 × 1, (**b**) 2 × 2, (**c**) 3 × 3, and (**d**) 4 × 4 PMUT arrays—comparison between finite-element simulations and the theoretical analytical solution.

As shown in Figure 5, comparison between the theoretical model and simulation results reveals that for different arrays, the theoretical predictions and the simulated data generally agree: the near-field exhibits oscillatory fluctuations due to multi-source interference, while in the far field, the sound-pressure amplitude decreases monotonically with propagation distance. As the array size increases from 1 × 1 to 4 × 4, the difference between theory and simulation grows unevenly. The error for the 2 × 2 array is similar to that of a single element, suggesting that at this size, the effects of inter-element coupling and multi-wave interference on on-axis pressure remain relatively limited. However, for the 3 × 3 and 4 × 4 arrays, the near-field discrepancy increases significantly, indicating that with more elements, the complexity of wave superposition and the effects of structure–medium coupling become more prominent.

Further analysis shows that the discrepancy mainly happens within about 1–2 times the element pitch. In this region, near-field interference dominates and is very sensitive to phase synchronization among elements. However, the theoretical model assumes a uniform point-source phase and ignores inter-element mechanical coupling, which makes it hard to fully capture the small phase differences caused by coupling and boundary effects in real arrays; this is a main source of error. Overall, the theoretical model accurately reproduces the main interference extrema and the attenuation trend seen in the simulations, confirming the effectiveness of the Bessel-mode description and the point-source superposition approach. Still, some phase shifts and amplitude deviations occur in the near field; prediction accuracy can be improved by including inter-element coupling terms, refining the radiation-boundary approximation, or using a more detailed equivalent-source model.

**Figure 6:**
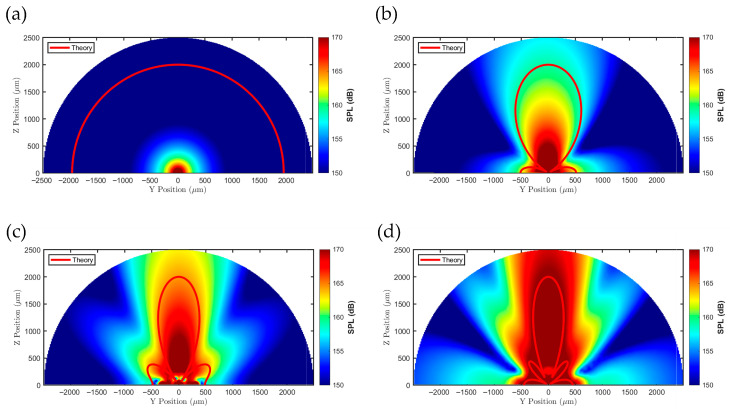
Theoretical directivity patterns and FEA-simulated YZ-plane SPL cross-sectional maps for the (**a**) 1 × 1, (**b**) 2 × 2, (**c**) 3 × 3, and (**d**) 4 × 4 PMUT arrays.

After scaling the theoretical directivity pattern to match the actual device geometry, overlaying it with the simulated sound-pressure map on the YZ-plane cross-section (Figure 6) shows that the high-SPL region of the predicted main lobe and the low-SPL regions corresponding to nulls closely match the simulation in terms of spatial locations. This provides an intuitive validation that the theoretical model accurately represents the primary radiation direction of acoustic energy. The figure also offers a quantitative comparison between the two across the full angular range (0–360°) and highlights angular intervals with lower agreement, such as minor deviations at certain sidelobes.

**Figure 7:**
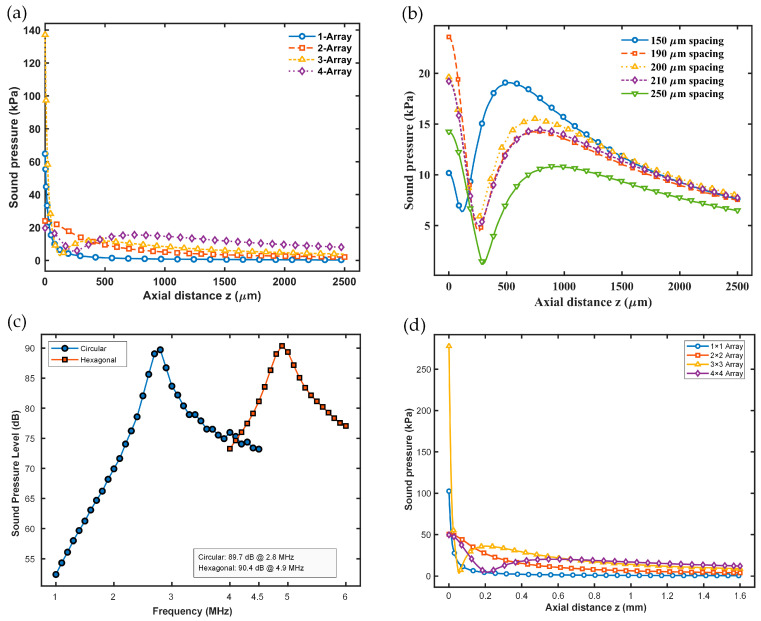
(**a**) On-axis sound-pressure decay for different circular arrays. (**b**) On-axis sound-pressure decay of 4 × 4 circular arrays with different pitch distances. (**c**) On-axis sound-pressure level at 1 m for circular vs. hexagonal PMUTs. (**d**) On-axis sound-pressure decay for hexagonal arrays.

Meanwhile, Figure 7a compares the on-axis sound-pressure decay for different arrays. The results show that although a single element has a relatively high initial sound pressure, it decreases quickly with distance and does not produce a clear focusing effect or a “plateau” region. As the array size increases, the superposition of multiple sources enhances interference effects and improves the effective radiation capability; however, local cancellation can also occur due to phase inconsistencies or path-length differences, which can reduce the sound pressure at certain distances.

Additionally, this study investigates how the far-field on-axis sound pressure of a 4 × 4 array varies with element pitch. As shown in Figure 7b, the spacing between elements significantly affects both the amplitude and the interference pattern of the acoustic field. When the pitch is 200 μm, the far-field sound pressure reaches its maximum. Based on these results, 200 μm is selected as the membrane-to-membrane spacing for the circular array, representing one of the most optimal options under the current design conditions.

The hexagonal arrays were also studied in this work. For a regular hexagonal array, deriving a fully analytical solution is generally more difficult because the honeycomb pattern lacks regular orthogonal symmetry, and the complexity of inter-element acoustic coupling and vibration transmission increases. Consequently, it becomes challenging to apply the standard analytical modeling assumptions used for regular arrays of circular elements directly. In this work, the hexagonal sound source is defined as a regular hexagon inscribed in a circle with a radius of 50 μm (Figure 4b). Comparing the frequency responses of on-axis SPL for single circular and hexagonal PMUTs reveals a notable difference, showing how the shape of the radiator influences radiation efficiency, resonance modes, and energy superposition mechanisms. As shown in Figure 7c, the hexagonal PMUT produces a higher far-field acoustic output: its SPL features a sharp resonance peak of 90.4 dB at 4.9 MHz, while the main resonance peak of the circular PMUT occurs at 2.8 MHz with 89.7 dB. Compared to a circular radiator, it has a higher resonance frequency and is thus more suitable for high-frequency ultrasound applications. Additionally, a 90 μm pitch is selected for the hexagonal array because this value offers higher far-field transmitting sensitivity; it is used for the subsequent array design and comparison.

To precisely assess the transmitting sensitivity of the hexagonal array, systematic finite-element modeling and simulations of hexagonal PMUT arrays were performed in COMSOL Multiphysics. During these simulations, the same material parameters, excitation conditions, and acoustic boundary conditions used for the circular PMUT arrays were applied (e.g., a hemispherical acoustic water domain and radiation boundary conditions), ensuring a reliable comparison. As shown in Figure 7d, the hexagonal array exhibits higher sound-pressure-transmitting sensitivity than the circular array (Figure 7a) of the same size in both the near-field and far-field regions, with a more noticeable advantage near the resonance frequency. This finding further confirms the potential of hexagonal arrays for high-performance acoustic device applications.

### 4.3. Study of the Dynamic Characteristics of PMUTs

The diaphragm’s dynamic properties serve as the essential link between electrical excitation, mechanical vibration, and acoustic radiation. Its resonance frequency, damping traits, and coupling efficiency directly affect the array’s frequency response, focusing capacity, and energy efficiency. The diaphragm is activated by a voltage pulse applied to the top electrode, as explained below:

(12) $$V_{0}=1\times e^{{\left(-f(t-3T)\right)}^{2}}\times \sin(2\pi ft).$$

We present the formula for the average displacement of a damped circular membrane, *Z_avg_*, as follows [32]:

(13) $$Z_{avg}(t)=\frac{\int_{0}^{t}F_{piezo}(t-h)e^{-\gamma h}\sin\omega_{1}h\cdot dh}{(m_{plate}+m_{aco})\omega_{1}}.$$

Here, *F_piezo_* denotes the total driving force acting on the diaphragm generated by the piezoelectric layer via the inverse piezoelectric effect, and it is related to the applied voltage *V* and the piezoelectric bending moment *M_piezo_*. *ω*_1_ is the damped natural angular frequency of the system (slightly lower than the undamped natural frequency). The maximum displacement of a circular membrane is approximately three times its average displacement [32]. In (13), *m_plate_* is the diaphragm mass, and *m_aco_* is the added acoustic mass. In this study, the structural parameters calculated in Table 9 are used. Here, *K_plate_* is the equivalent stiffness coefficient of the diaphragm, *b_total_* is the total damping coefficient, *Υ* is the attenuation constant. From the above equations, the maximum velocity of the average dynamic displacement is derived as 0.0159 m/s. In addition, we provide the expression for the far-field sound pressure as follows [32]:

(14) $$P_{ff}\left(s\right)=P_{0}\frac{R_{0}}{s}=\rho_{aco}c_{aco}u_{avg}\frac{A}{\lambda s}=\frac{\rho_{aco}}{8}\frac{\omega }{s}D^{2}u_{avg}$$

Here, *s* is the perpendicular distance to the diaphragm, *P*_0_ is the theoretical surface pressure, $\rho_{aco}$ is the density of the acoustic medium, *c_aco_* is the sound speed in the acoustic medium, *u_avg_* is the average particle velocity over the effective radiating surface area, *R*_0_ is the Rayleigh distance associated with the effective radiating area, *A* is the diaphragm surface area, and *λ* is the wavelength of the pressure wave.

**Table 9:** Equivalent diaphragm performance parameters.

*R* (μm)	*R_d_* (μm)	*T* (μm)	*∂M_piezo_/∂V* (N/V)	*Υ* (rad/s)	*ω*_1_ (rad/s)
50	28	4.22	3.09 × 10^−6^	1.8 × 10^6^	1.82 × 10^7^
*K_plate_* (N/m)	*M_acou_* (kg)	*M_plate_* (kg)	*b_total_* (kg/s)	*F_piezo_* (N)	*D_m_* (N·m)
1.83 × 10^5^	2.63 × 10^−10^	2.9 × 10^−10^	2.05 × 10^−3^	5.7 × 10^−5^	7.6 × 10^−7^

Thus, the sound pressure at 100 mm is estimated to be 3.49 Pa. In the simulation, the transient peak sound pressure at 1 mm is 296 Pa; by fitting it with a 1/*s* decay curve [32], the sound pressure at 100 mm is calculated as approximately 2.96 Pa. The theoretical prediction and simulation results agree well.

**Figure 8:**
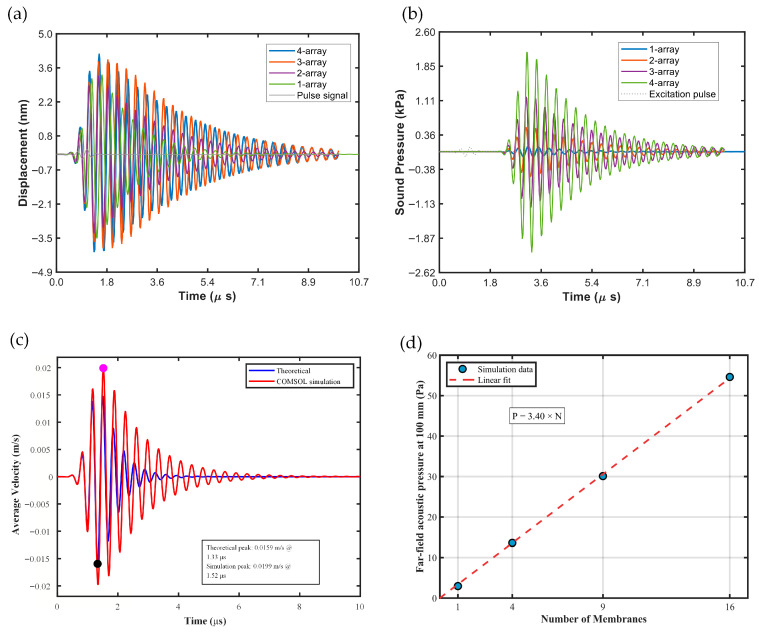
(**a**) Transient displacement of the diaphragm center for 1 × 1, 2 × 2, 3 × 3, and 4 × 4 arrays. (**b**) Transient on-axis sound-pressure attenuation at 2.5 mm for these arrays. (**c**) Time evolution of the average displacement for the 1 × 1 array. (**d**) Predicted sound pressure at 100 mm for the 1 × 1, 2 × 2, 3 × 3, and 4 × 4 arrays.

As shown in Figure 8c, based on the previously calculated equivalent diaphragm parameters, we further predict the time-domain evolution of the dynamic average velocity. In the single-cell model comparison, the theoretical model indicates that the average velocity peaks at approximately 1.33 μs with a value of 0.0159 m/s; in contrast, COMSOL transient simulations show that the peak occurs around 1.52 μs, reaching 0.0199 m/s. Discrepancies in both peak timing and magnitude may result from the numerical model accounting more thoroughly for factors such as material damping, acoustic radiation impedance, and boundary and coupling conditions, leading to deviations in effective damping and inertial loading compared to the simplified theoretical model. Overall, the two results follow similar trends and are within the same order of magnitude, supporting the validity of the proposed theoretical model for rapid prediction and indicating that additional coupling or weak nonlinear effects may be present in practical devices. This provides a foundation for further PMUT structural parameter optimization and refinement of the simulation model.

Meanwhile, this study simulated the transient peak displacement of different arrays (Figure 8a) and the transient on-axis sound-pressure decay at 2.5 mm (Figure 8b), where the wave peak pressure has already attenuated significantly at this boundary. Among them, the *R*_0_ of the 4 × 4 array is 0.927 mm, indicating that the region at 2.5 mm is already in the far-field region. A 1/*s* fitting model was applied to the decay curves to extrapolate the far-field peak sound pressure of each array (Figure 8d).

The predicted sound pressures at 100 mm for the four arrays are 2.96 Pa, 13.6 Pa, 30.1 Pa, and 54.6 Pa, respectively. A linear function was used to fit the predicted sound-pressure values at 100 mm for the 1 × 1, 2 × 2, 3 × 3, and 4 × 4 arrays, resulting in a relationship of 3.40 × *N*. It is evident that, in the far field, the output sound pressure is directly proportional to the number of diaphragms.

To benchmark the performance of the proposed PMUT design, we compared the simulated transmitting sensitivity with three recently reported PMUT arrays based on PZT and AlN thin films [33,34,35]. Since the acoustic pressure was measured or simulated at different distances in these studies, we normalized all sensitivity values to a standard distance of 100 mm using the inverse-distance law. The Rayleigh distance for devices in various literature was calculated according to the Rayleigh distance criterion, and the original measurement distance is much greater than the Rayleigh distance, indicating that the measurement point is in the far-field region and the sound pressure follows the spherical wave attenuation law. As summarized in Table 10, the proposed circular PZT PMUT exhibits a normalized theoretical sensitivity of 2.96 Pa/V. This value represents a notable improvement over the referenced experimental results. Specifically, our design achieves approximately 6.8 times the sensitivity of the I-shaped PZT PMUT [33] and 2.3 times that of the standard PZT array [35]. Additionally, the design shows a 2.2-fold increase compared to the AlN-based PMUT [34]. This superior acoustic output validates the efficacy of the proposed design strategy, in which accurate material parameters derived from first principles guide the geometric optimization to achieve enhanced electromechanical conversion. It is worth noting that the performance gap between our simulation and the references [33,34,35] can be partially attributed to fabrication-induced imperfections, such as residual stress and material defects, which are inevitable in practical processing.

## 5. Conclusions

This work addresses two key challenges in PMUT array design—inaccurate material parameters and inefficient large-array simulation—by establishing a design framework that integrates first-principles calculations with theoretical acoustic-field modeling. First-principles calculations provide the full-tensor properties of PZT and ScAlN, confirming PZT’s superior transmitting sensitivity and electromechanical coupling efficiency due to its higher piezoelectric stress constants. These intrinsic parameters serve as a physically consistent and traceable baseline that mitigates the uncertainties associated with empirical software libraries derived from bulk ceramics, thereby providing a more reliable foundation for device design. To overcome the simulation bottleneck, an efficient acoustic-field prediction model based on thin-film vibration modes and the Helmholtz–Kirchhoff integral is developed. This model maintains accuracy while avoiding the computational limits of full-wave finite-element simulations for large arrays, enabling rapid evaluation of acoustic fields and directivity, thereby shortening design cycles. Integrated analysis shows that a hexagonal array layout enhances far-field sound pressure and yields a more favorable high-frequency acoustic distribution than conventional circular arrays. In summary, this modeling approach using intrinsic parameters enhances the physical fidelity of PMUT design and provides an efficient theoretical and engineering tool for developing next-generation high-performance, low-power ultrasonic arrays in portable medical imaging and sensing.

## Figures and Tables

**Table 1:** Calculated elastic stiffness constants *C_ij_* (in GPa) of the tetragonal PZT supercell.

Index	1	2	3	4	5	6
1	231.96	88.02	83.87	−1.42	1.74	4.53
2	88.02	231.64	84.16	−2.00	1.85	4.60
3	83.87	84.16	95.23	−1.40	2.94	12.18
4	−1.42	−2.00	−1.40	48.39	2.06	1.68
5	1.74	1.85	2.94	2.06	50.18	0.98
6	4.53	4.60	12.18	1.68	0.98	75.40

**Table 2:** Calculated piezoelectric stress constants *e_ij_* (in C/m^2^) of the tetragonal PZT supercell.

Index	1	2	3	4	5	6
1	0.03	0.05	−0.07	−0.06	23.57	0.01
2	0.10	0.04	−0.05	24.35	−0.04	0.04
3	1.24	1.24	4.31	0.00	0.00	0.00

**Table 3:** Calculated relative dielectric constants *ε_ij_* of the tetragonal PZT supercell.

Index	1	2	3
1	875.43	−1.85	0.059
2	−1.847	902.34	−0.051
3	0.059	−0.05	24.07

**Table 4:** Calculated elastic stiffness constants *C_ij_* (in GPa) of wurtzite Sc_0.25_Al_0.75_N.

Index	1	2	3	4	5	6
1	279.2	135.7	135.5	0.0	0.0	0.0
2	135.7	275.6	135.3	0.0	0.0	0.0
3	135.5	135.3	171.7	0.0	0.0	0.0
4	0.0	0.0	0.0	63	0.0	0.0
5	0.0	0.0	0.0	0.0	62.4	0.0
6	0.0	0.0	0.0	0.0	0.0	72.3

**Table 5:** Calculated piezoelectric stress constants e*_ij_* (in C/m^2^) of wurtzite Sc_0.25_Al_0.75_N.

Index	1	2	3	4	5	6
1	0.00	0.00	0.00	0.00	−0.32	0.00
2	0.00	0.00	0.00	−0.32	0.00	0.00
3	−0.68	−0.68	1.97	0.00	0.00	0.00

**Table 6:** Calculated relative dielectric constants ε*_ij_* of wurtzite Sc_0.25_Al_0.75_N.

Index	1	2	3
1	9.74	0.00	0.00
2	0.00	9.74	0.00
3	0.00	0.00	12.05

**Table 7:** Comparison of calculated material properties (this work) with literature values for ScAlN and PZT.

Material	Parameter	This Work (DFT-PBE)	Literature (DFT/Exp)	Reference
AlScN	*C*_33_	171.7	150–400	[25,26]
	*e*_33_	1.97	1.50–10	[25,26]
PZT	*C*_33_	95.23	~195.72	[27]
	*e*_33_	4.31	~4.81	[28]

**Table 8:** Sensitivity of the on-axis Sound Pressure Level (SPL) at 1 m to variations in the piezoelectric stress constant *e*_33_ for the PZT-based PMUT.

Variation in *e*_33_	Variation in *e*_33_ (C/m^2^)	Resulting Change in SPL at 1 m (dB)
+20%	5.18	+2.3
+10%	4.74	+1.2
0%	4.31	0.00
−10%	3.88	−1.6
−20%	3.45	−3.3

**Table 10:** Comparison of transmitting sensitivity between the proposed PMUT and recently reported works.

Material	Frequency	*R*_0_	Reported Sensitivity (Distance)	Normalized Sensitivity (@ 100 mm)	Reference
PZT (Circular)	2.8 MHz	14.8 μm	2.96 Pa/V (100 mm)	2.96 Pa/V	This work
PZT (I-Shaped)	0.76 MHz	150 μm	2.18 Pa/V (20 mm)	~0.44 Pa/V	[33]
AlN (Circular)	3.58 MHz	14 μm	9.07 Pa/V (15 mm)	~1.36 Pa/V	[34]
PZT (Circular)	0.5 MHz	5.33 mm	4.24 Pa/V (30 mm)	~1.27 Pa/V	[35]

## Data Availability

The datasets presented in this article are not readily available because the data are part of an ongoing study. Requests to access the datasets should be directed to xudacheng@suda.edu.cn or hexingli@suda.edu.cn.
